# An open dataset and machine learning algorithms for Niacin Skin-Flushing Response based screening of psychiatric disorders

**DOI:** 10.1186/s12888-025-07196-2

**Published:** 2025-08-04

**Authors:** Xuening Lyu, Rimsa Goperma, Dandan Wang, Chunling Wan, Liang Zhao

**Affiliations:** 1 Graduate School of Advanced Integrated Studies in Human Survivability, Kyoto University, Kyoto, Japan; 2 Bio-X Institutes, Shanghai Jiao Tong University, No. 1954, Huashan Road, 200030 Shanghai, China

**Keywords:** Niacin Skin-Flushing response (NSR), Artificial intelligence in mental health, Mental disorders diagnostics, Machine learning algorithms for healthcare, Device-Independent biomarkers

## Abstract

**Background:**

Niacin Skin-Flushing Response (NSR) has emerged as a promising objective biomarker for the precise diagnosis of mental disorders. However, its diagnostic potential has been constrained by the limitations of traditional statistical approaches. The advent of Artificial Intelligence (AI) offers a transformative opportunity to overcome these challenges. This study presents a novel contribution to the field by establishing an open-access dataset and developing advanced AI-driven tools to enhance the diagnostic accuracy of psychiatric disorders through NSR analysis.

**Methods:**

This study introduces the world’s first open dataset specifically developed for AI studies of Niacin Skin-Flushing Response (NSR), a physiological biomarker associated with mental illnesses including depression, bipolar disorder, and schizophrenia. Leveraging this dataset, we developed an advanced Machine Learning (ML) approach designed for the broad diagnosis of mental disorders. Distinct from prior studies which are often limited to First Episode Schizophrenia and depend on specific devices, our approach champions device independence. The core of our methodology involves a novel algorithm featuring an Efficient-Unet based Deep Learning model for the precise segmentation of NSR areas. This segmentation is significantly enhanced by runtime data augmentation and trained on a robust train/validation/test dataset split. Subsequently, a Support Vector Machine (SVM) method is employed for psychiatric disorder classification utilizing feature vectors extracted from the segmentation of NSR areas with a 3-scale quantization. The SVM training incorporates 5-fold cross-validation, Synthetic Minority Over-sampling Technique (SMOTE) for managing class imbalance, and hyperparameter tuning to optimize balanced accuracy.

**Results:**

The established dataset comprises 600 high-quality NSR images from 120 individuals, encompassing a diverse cohort of healthy controls and patients with various mental illnesses. The developed AI tools offer an objective, swift, and highly accurate approach that is demonstrably independent of the diagnosed condition or the specific device used for image acquisition. Comparative results demonstrate that the ML-based diagnostic approach achieves a sensitivity ranging from 60.0 to 65.0% and a specificity from 75.0 to 88.3% across various types of illnesses, further underscoring its broad applicability and device independence.

**Conclusions:**

This research conclusively demonstrates the significant potential of advanced AI tools in achieving precise diagnosis of psychiatric disorders, potentially surpassing human capabilities in both speed and accuracy. With the provision of the proposed open dataset and the introduction of novel methodologies, this study marks substantial progress in developing an objective and accurate NSR-based screening process for a wide spectrum of psychiatric disorders. Its enhanced applicability and independence from specific devices hold profound potential to substantially advance mental health diagnostics and contribute to improved patient outcomes globally.

**Supplementary Information:**

The online version contains supplementary material available at 10.1186/s12888-025-07196-2.

## Key messages

**What is already known on this topic**

Niacin Skin-Flushing Response (NSR) is known to be a promising approach to screening psychiatric disorders. However, existing methods depend heavily on human efforts in segmenting the NSR area from pictures. On the other hand, Artificial Intelligence (AI) based methods have demonstrated the possibility to improve the precision of image segmentation and classification. However, existing AI-based methods depend on special equipment and lack an open dataset for a fair benchmark.

**What this study adds**

We introduce a first-of-its-kind, open NSR dataset and a precise, device-independent AI diagnostic approach. This broadens the applicability of NSR-based biomarkers and demonstrates promising diagnostic accuracy across various mental illnesses.

**How this study might affect research**,** practice**,** or policy**

Without an open dataset, any AI-based method is limited because it depends on the dataset in developing. Therefore, this study can guide future AI-based NSR research and boost the accuracy and accessibility of mental health diagnostics. It can also inform policy changes to incorporate AI in mental health diagnostics, enhancing patient outcomes.

**Supplementary Information:**

The online version contains supplementary material available at 10.1186/s12888-025-07196-2.

## Introduction

Depression (DP), bipolar disorders (BP), and schizophrenia (SZ) are significant psychiatric disorders (PD) that affect millions of people globally [1–3]. Due to the involvement of extensive pathological processes and diverse clinical manifestations, the diagnostic procedures for these psychiatric disorders are typically complex and time-consuming [1–3]. Identifying objective auxiliary diagnostic markers holds significant promise in addressing this clinical challenge.

Despite this necessity, the search for objective diagnostic markers is hampered by the limited understanding of the causes and mechanisms underlying mental disorders. This study aims to address this gap by investigating the Niacin Skin-Flushing Response (NSR), a physiological response that has shown promise as a potential diagnostic marker for psychiatric disorders. Extensive research has demonstrated that individuals with psychiatric disorders, such as SZ, exhibit significantly attenuated responses in NSR tests, necessitating substantially higher niacin concentrations to elicit a comparable skin-flushing relative to healthy controls (HCs) [1–3]. Emerging evidence has consistently revealed impaired NSR in patients with DP and BP [2, 3], which are two types of psychiatric disorders that share overlapping negative symptoms with SZ [4].

The promising diagnostic utility of NSR has catalyzed extensive research efforts and spurred the development of innovative methodologies for its precise evaluation and quantification. Earlier niacin tests relied on oral administration [1], a process susceptible to variation due to niacin intake. Subsequent studies improved this method by utilizing filter paper soaked in a niacin solution [5, 6]. However, the assessment of NSR remains predominantly limited to a 4-point grading scale [3, 6, 7]. While numerous studies have attempted to develop fully quantitative approaches for evaluating flushing responses - such as utilizing laser Doppler to measure blood flow velocity [8, 9], employing optical reflectance spectroscopy to assess hemoglobin concentration changes [10, 11], or calculating erythema area on the arm [12] - these methods are inherently constrained by their heavy reliance on the specificity of the testing instruments and consumables used. Additionally, these methods remain quite subjective, as they require human effort to quantify the NSR. The advent of Artificial Intelligence (AI) technologies has paved the way for more objective methodologies in NSR assessment [13, 14]. Yet, existing AI methods often require specific devices and have predominantly been applied to single mental disorder such as First Episode Schizophrenia (FES) [13]. Moreover, the literature lacks a publicly available dataset to compare different methods, thereby limiting the broader application of AI methods.

In this study, we aim to overcome these limitations by developing a generalized, device-independent AI algorithm that uses the NSR to diagnose various mental disorders. We have curated the first publicly available NSR dataset, consisting of 600 images from 120 individuals, including HCs and patients with mental disorders. We employed a U-net based Deep Learning algorithm for quantifying the NSR from images and a Support Vector Machine (SVM) for classifying psychiatric disorders. Based on the dataset and the algorithms, we aimed to establish a generalized, device-independent methodology for mental disorder diagnostics. By transcending dependence on specific devices, our approach has the potential to democratize mental health diagnostics and significantly improve patient outcomes by making the benefits of AI accessible to a wider range of psychiatric disorders.

## Methods

### Experimental design

**Fig. 1:**
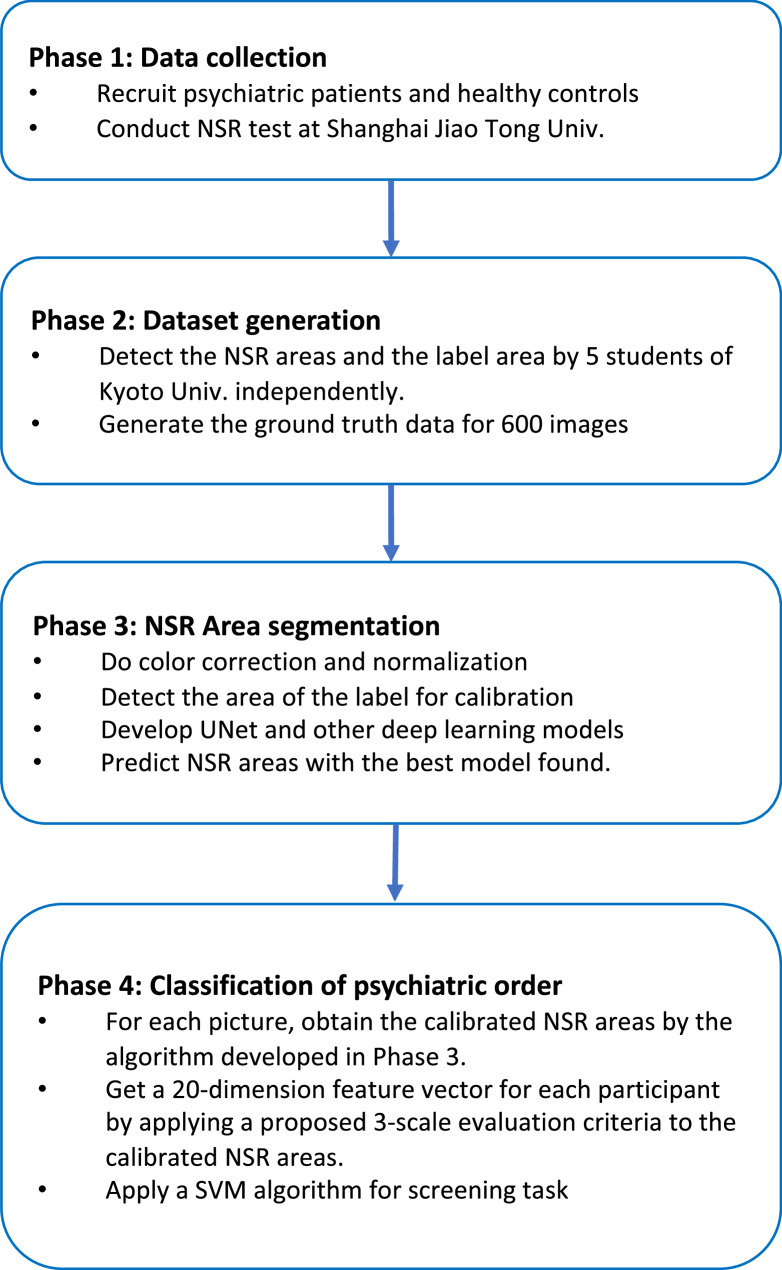
The flowchart of this study

The data used in this study were collected at Shanghai Jiao Tong University. For the acquired image data, we applied image segmentation algorithms to detect the NSR area and quantify the NSR degree. The detection process includes image pre-processing such as color correction and normalization, area detection by artificial neural network (ANN). Then we utilized an SVM model to classify the types of mental disorders. To evaluate the performance of the classification, we compared two different screening methods. Figure 1 shows the entire experimental design. A tabular summary of the keywords has been included in Supplementary Table 1, providing a concise overview of the key concepts and terminologies used in the study.

### Data collecting

#### Inclusion criteria

Participants were recruited from various hospitals in China and met specific inclusion criteria. They were free from severe neurological diseases, significant brain trauma, substance dependence, and had no history of using non-steroidal or steroidal anti-inflammatory drugs in the week leading up to the NSR test. Participants were not suffering from any skin or immune system diseases and were not pregnant at the time of the study. Informed consent was obtained from all participants before the start of the study. The study adhered to the guidelines of the local ethics committee for data collection. The study involved a total of 773 participants, divided into four distinct groups. The detail of the data collection is described in Wang et al. [6]. For completeness, we provide a summary in Supplementary Fig. 1. All averages are reported with an associated standard deviation (SD).

#### Niacin test

For the NSR test, we employed a specially designed patch with a sandwiched structure. This patch consists of a piece of filter paper covered by foam tape with four 1 cm diameter holes. The back of the filter paper was coated with thin, skin-friendly gels with holes aligned to those on the tape. The NSR test was conducted as follows: (1) A niacin patch, featuring four holes, was prepared. Each hole was then filled with a distinct concentration (0.1, 0.01,0.001, and 0.0001 M) of the methyl nicotinate solution (AMN, C_7_H_7_NO_2_, 99%, Sigma-Aldrich). (2) A standardized label with the participant’s identification number and the niacin patch were affixed to the participant’s forearm for one minute, and a photograph was taken. Subsequently, the niacin patch was removed, but the label remained on the arm. (3) Photographs were taken every minute for the next 20 min. To provide a more intuitive illustration of the NSR test process, we have created a flowchart of the niacin test, as shown in Supplementary Fig. 1. For each photograph, researchers from Shanghai Jiao Tong University conducted a 4-point scale assessment of the NSR-induced flushing (refer as Manual score), based on the following criteria: 0 = no erythema, 1 = incomplete erythema, 2 = complete erythema within the defined area of the patch, and 3 = erythema beyond the defined area of the patch. Photographs of the subjects taken at the 1-minute, 5-minute, 10-minute, 15-minute, and 20-minute marks were utilized for subsequent open dataset analysis.

### Open dataset

Based on the collected data, we constructed the world’s first NSR dataset, which comprises the judgements of five independent individuals on the NSR area derived from an analysis of 600 photographs (Supplementary Fig. 2). The NSR dataset comprises photographs of subjects from four distinct groups. To ensure data balance, we randomly selected 20 subjects from each of DP, BP, and SZ groups (totaling 60 subjects), along with 60 subjects from the HC group. Subsequently, we manually extracted the regions containing both the labels and the targeted skin area for further analysis.

**Fig. 2:**
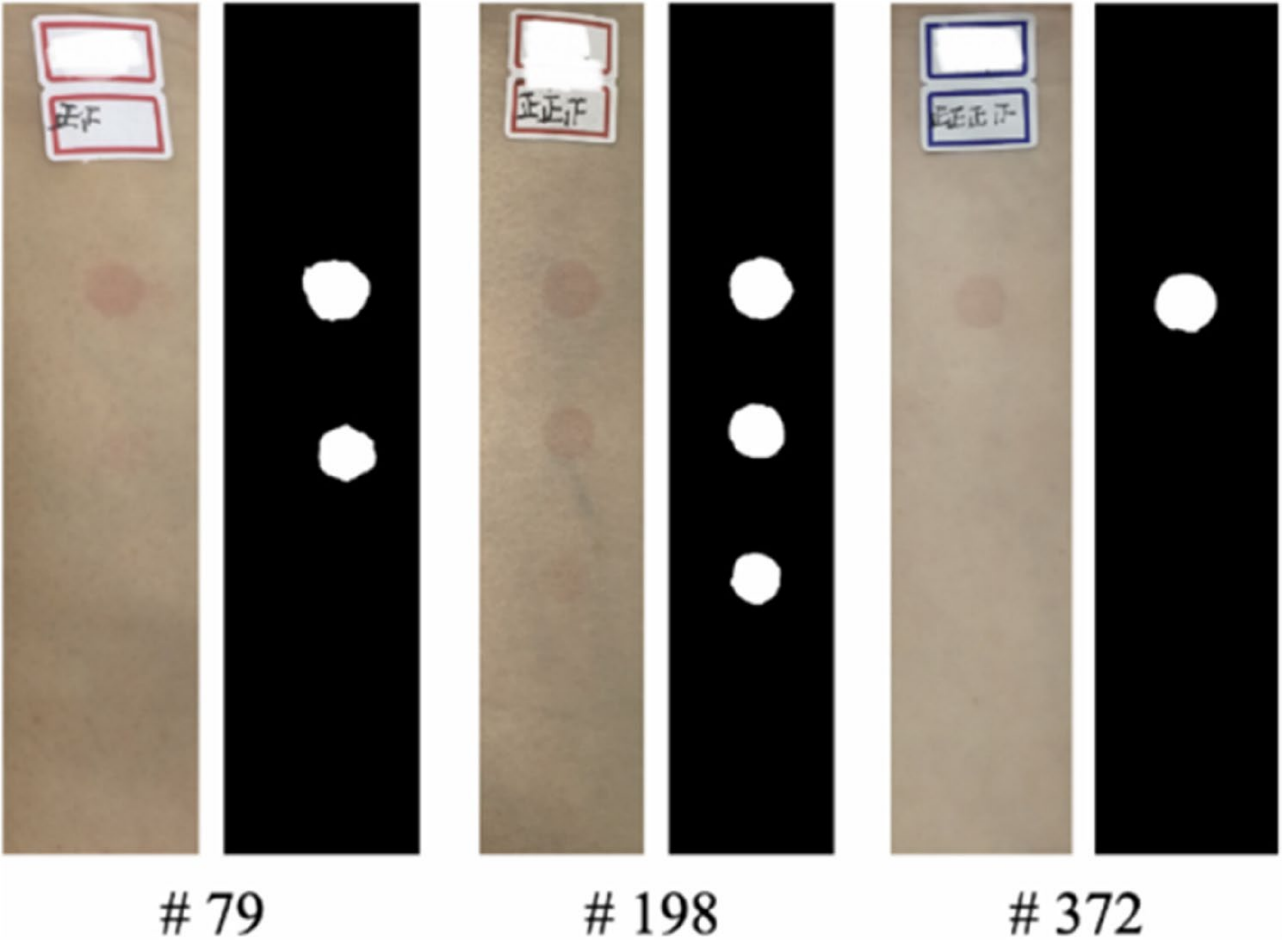
Sample images from the dataset. The image on the left of each group is the skin photo; the image on the right represents the flushing areas obtained by manual annotation

To get the ground-truth of annotated images, we manually mark the NSR area in the images as follows. Since the result depends on the equipment and the color vision ability of the person who marks it, we enlisted five students who have no color blindness and asked them to annotate all the pictures with the same type of iPad and Apple Pencil. Moreover, we observed that different students may mark differently for the boundary of NSR area. To increase the reliability of annotation, we classified a pixel to be in the NSR area if and only if at least two students annotated it so. The label serves as a useful tool for length calibration as we lacked information regarding the absolute size of flushing areas. The dataset comprises 600 images of skin and their corresponding binary masks. Each skin picture captures the niacin application area and an attached label on the arm. The 4-point scale assessment results conducted by researchers at Shanghai Jiao Tong University were also included in a reference file (info.csv) within the database. For illustrative examples of the dataset’s photographs, showcasing both the captured skin images and the manually annotated flushing areas, refer to Fig. 2.

## Data analysis

The data analysis pipeline was bifurcated into two primary stages: precise NSR area detection via Deep Learning-based segmentation, followed by Machine Learning (ML) classification for psychiatric disorder screening.

### Image pre-processing and data augmentation

To prepare the raw NSR images for analysis and enhance model performance, a series of pre-processing and augmentation steps were applied. Initially, color correction was performed using the Perfect Reflector Method, leveraging the white section of the included label as a standardized white reference to mitigate color inconsistencies arising from diverse acquisition devices and varying lighting conditions. Concurrently, an image size normalization step was executed by utilizing the consistent real-life size of the arm label as a reference, thereby compensating for variations in camera-to-arm distances across different tests. After these initial corrections, all images were uniformly resized to 512 × 128 pixels, a rectangular aspect ratio optimized for the input requirements of the subsequent Deep Learning models.

To significantly bolster the robustness and generalization capabilities of our segmentation models, runtime data augmentation was extensively implemented during the training phase using the Albumentations library (https://albumentations.ai/). This dynamic augmentation strategy involved applying various transformations such as random rotations, shifts, scaling, flips (horizontal/vertical), and controlled adjustments to brightness, contrast, and saturation. This approach allowed the models to learn more invariant and discriminative features, reducing overfitting and improving performance on unseen data.

### NSR area detection (segmentation)

For the critical task of identifying and segmenting the subtle NSR areas, which often exhibit low contrast and indistinct boundaries, an advanced Deep Learning approach was adopted. This project explored multiple state-of-the-art segmentation models to identify the most effective architecture for the task. We chose the following Deep Learning models Vit-Unet, Resnet152-Unet, Effb5-Unet, Mob-Deeplab, Mob-DeeplabPlus, Resnet152-Unet++, and plain UNet because they are encoder-decoder based semantic segmentation models known for efficiency and robust performance in medical image analysis, particularly with limited computational resources and data. Their proven capabilities in producing dense pixel-wise predictions, their relative computational efficiency for training on a new dataset, and their interpretability in identifying precise regions made them suitable for our initial objective of establishing a robust and efficient baseline.

We remark that while Mask R-CNN and DeepLabV3 + are prominent architectures in the field of computer vision, they were not the primary focus for this particular segmentation task for a few key reasons. Mask R-CNN, primarily an instance segmentation model, is optimized for detecting and segmenting individual objects within an image. Our task, however, is a semantic segmentation problem, where we are interested in identifying a single, continuous region (the flushed area) rather than individual instances. Applying Mask R-CNN directly would be an overkill and potentially less efficient for this specific problem type, possibly leading to unnecessary computational overhead and complexity in post-processing to consolidate instances into a single semantic region. DeepLabV3+, on the other hand, is a powerful semantic segmentation model but often leverages dilated convolutions and large receptive fields, which can be computationally intensive and might require larger datasets or more aggressive training strategies to generalize effectively on fine-grained segmentation tasks like NSR, especially given the subtle boundaries. Our objective is to establish a robust and efficient baseline, making encoder-decoder architectures, particularly U-Net variants known for their efficacy in biomedical image segmentation, more suitable for a direct comparative evaluation on our curated dataset.

Among the evaluated models, the Efficient-Unet (Effb5-Unet) architecture consistently demonstrated superior performance in accurately segmenting the NSR areas from the corrected and augmented images. The comprehensive dataset was divided into distinct sets for training, validation, and testing at the patient level, ensuring that no patient’s data appeared in more than one set. Since there are 120 unique participants, this rigorous partitioning involved 90 of them for training, 10 for validation, and 20 for independent testing. Models were trained using standard optimization protocols, with performance monitored via common metrics including Dice coefficient and Intersection over Union (IoU). We employed no post-processing since the results were good enough and we wanted to directly compare the Deep Learning models only.

### NSR quantification and screening approaches

Following the NSR area detection, we explored various methods to quantify the NSR and subsequently screen for psychiatric disorders. Previous studies have utilized a 4-point scale for measuring NSR degrees. Recognizing the inherent subjectivity of manual annotation, we developed a more objective 3-scale, derived from the automatically detected NSR areas and their correspondence to manually assigned scores (see the following). Besides this score-based approach, we also applied a direct screening method utilizing the raw detected NSR areas, bypassing discrete score assignment. A comparative analysis of these methods was performed to corroborate the efficacy of our proposed objective quantification.

### NSR area scoring (objective 3-scale)

For each detected region, we calculate the normalized area, A_norm_​, as the ratio of the detected NSR area A_detected_​ to the corresponding labeled area A_label_​ (the area of the label attached to the arm), i.e.,

A_norm_​ = A_detected_/​​ A_label_.

We then analyzed the mean value, variance, and standard deviation of the normalized area distributions corresponding to each human score. The observed results were as follows: For score 0, the mean value, variance, and the standard deviation were 0.0648, 0.0229, and 0.1515, respectively. For score 1, they were 0.1535, 0.0363, and 0.1907, respectively. For score 2, they were 0.1661, 0.0217, and 0.1477, respectively. And for score 3, they were 0.1665, 0.0291, and 0.1706, respectively. While the distributions of the normalized areas vary across different scores, there is a considerable overlap and variance. Consequently, we established the next objective 3-scale scoring system based on the above observations:

Score 0 if A_norm_ < 0.1091; Score 1 if 0.1091 ≤ A_norm_ < 0.1598; and Score 2 otherwise.

### Feature extraction for classfication

For each participant, based on the NSR areas detected by the Efficient-Unet model, we created two distinct types of 20-dimensional feature vectors for classification, each capturing the scores of normalized sizes of the four NSR areas at five critical time points (1st, 5th, 10th, 15th, and 20th minute) post-application. Firstly, we established the objective 3-scale score-based feature vector as explained before. Each element represented the objectively derived 3-scale score (0, 1, or 2) for a specific niacin patch concentration at a given time point. Secondly, we established the direct NSR area feature vector. Each element represented the normalized NSR area A_norm_ directly for a specific niacin patch concentration at a given time point. This vector comprehensively captured the dynamic and concentration-dependent physiological response with high granularity and objectivity, without discrete score assignment.

### Psychiatric disorder screening (classification)

The final stage of the analysis involved classifying participants into specific diagnostic groups based on their extracted NSR feature vectors. A Support Vector Machine (SVM), a robust supervised learning model well-suited for high-dimensional data, was selected for this classification task. To ensure the SVM’s performance was optimized and robust against potential biases, several advanced techniques were employed.

#### 5-Fold cross-validation

The entire dataset of feature vectors was subjected to 5-fold cross-validation. In each iteration, 80% of the data served as the training set, and the remaining 20% as the test set, ensuring a comprehensive and reliable evaluation of the model’s generalization capabilities. This process was iteratively performed until each subset had been used as the test set once.

#### SMOTE for class imbalance

Given the inherent class imbalances often present in clinical datasets, the Synthetic Minority Over-sampling Technique (SMOTE) was applied to the training data within each cross-validation fold. This algorithm synthesized new minority class samples, thereby balancing the class distribution and preventing the SVM from being unduly biased towards the majority healthy control group.

#### Hyperparameter tuning

Extensive grid search was performed within each cross-validation fold to ascertain the optimal hyperparameters for the SVM model (e.g., kernel function (radial basis function was generally preferred), ‘C’ regularization parameter, and ‘gamma’ for RBF kernel). This meticulous tuning aimed to maximize the balanced accuracy of the classifier, a crucial metric for imbalanced datasets, thereby pushing the SVM’s predictive performance to its empirical limit.

The final classification performance was evaluated using standard metrics including overall accuracy, as well as class-specific precision, recall (sensitivity), and specificity, across various binary classification tasks (e.g., HC vs. Depression, HC vs. Schizophrenia, HC vs. Bipolar Disorder).

## Results

### Analysis of NSR response

Study on the NSR with a manual 4-scale scoring system revealed significant differences in mean NSR scores between the psychiatric patient groups and the Healthy Control (HC) group. However, no significant differences were observed among the psychiatric groups themselves. Niacin tests conducted with 0.0001 M AMN were generally omitted from analysis due to a prevalent lack of NSR induction at this concentration. These observations confirmed that NSR areas tend to develop more slowly in psychiatric patients compared to healthy controls, thereby reinforcing the need for more objective and precise NSR area detection and effective screening methodologies.

### Results of NSR area detection (segmentation)

This project implemented an advanced segmentation pipeline. Image data was adapted to a rectangular size of 512 × 128 pixels to best suit the input requirements. To significantly enhance model generalization and robustness, runtime data augmentation was dynamically applied using the Albumentations library during training. For robust model training and evaluation, the dataset was strategically split at the participants level, ensuring distinct sets for different phases: 90 for training, 10 for validation, and 20 for final testing. A comprehensive evaluation of multiple advanced Deep Learning segmentation models was conducted. The performance of these models, quantified by Dice and IoU metrics on the test set, is summarized in Table 1.

**Table 1:** Performance metrics for NSR area detection models (Dice and IoU)

Model	Dice (%)	IoU (%)
Vit – Unet	90.32	82.38
Resnet152-Unet	90.78	83.15
**Effb5-Unet**	**91.31**	**84.06**
Mob-Deeplab	90.22	82.24
Mob-DeeplabPlus	90.36	82.46
Resnet152-Unet++	89.73	81.4
UNet	86.23	75.89

As presented in Table 1, the Efficient-Unet (Effb5-Unet) architecture consistently achieved the best performance among the tested models, demonstrating an impressive Dice score of 91.31% and an IoU of 84.06%. It is an important methodological consideration that, for this study, post-processing was intentionally omitted for the segmented masks. This approach allowed for a direct assessment of the raw predictive capabilities of the Deep Learning segmentation models without external refinements.

### Results of psychiatric disorder screening (classification)

After image segmentation and quantification, different types of feature vectors were prepared for training a SVM classifier. To effectively mitigate the challenges posed by class imbalance within the dataset and to maximize diagnostic accuracy, SMOTE was judiciously employed within each fold of the 5-fold cross-validation. Furthermore, exhaustive hyperparameter tuning was performed to fine-tune the SVM model parameters, specifically aiming to optimize its balanced accuracy. The robustness and generalizability of the classification models were rigorously assessed through 5-fold cross-validation.

#### Performance of objective 3-scale score classification

We evaluated screening using feature vectors derived from our objectively calculated 3-scale scores. The quantitative results detailing the SVM-based classification performance for distinguishing Healthy Controls (HC) from specific psychiatric groups are summarized in Table 2.

**Table 2:** SVM classification performance using objective 3-scale score features for HC vs. specific psychiatric disorders (5-Fold CV balanced Accuracy)

Comparison	Best parameters found (Example)	Balanced accuracy	Sensitivity (recall for patient group)	Specificity (recall for HC)	Overall accuracy
HC vs. BP	smote_k_neighbors = 7, svc_C = 0.1, svc_gamma = 0.01, svc_kernel: rbf	0.68	0.6000 (BP)	0.7500 (HC)	0.71
HC vs. SZ	smote_k_neighbors = 5, svc_C = 10, svc_degree = 4, svc_kernel: poly	0.72	0.6500 (SZ)	0.7833 (HC)	0.75
HC vs. DP	smote_k_neighbors = 7, svc_C = 1, svc_degree = 2, svc_kernel: poly	0.74	0.6000 (DP)	0.8833 (HC)	0.81

#### Detailed precision and recall for objective 3-scale score classification

The classification reports further detail the precision, recall, and f1-score for each class. The classification reports provide detailed performance metrics for each diagnostic group in the three binary classification tasks. For HC vs. BP, the model achieved a precision of 0.85 and recall of 0.75 for HC, while BP classification yielded a precision of 0.44 and recall of 0.60. In the HC vs. SZ task, HC demonstrated a precision of 0.87 and recall of 0.78, whereas SZ exhibited a precision of 0.50 and recall of 0.65. Lastly, for HC vs. DP, the classifier showed strong performance for HC (precision: 0.87, recall: 0.88) and moderate performance for DP (precision: 0.63, recall: 0.60). These results highlight varying discriminatory power across diagnostic categories, with consistently higher precision and recall for HC compared to psychiatric conditions.

#### Detailed confusion matrices for objective 3-scale score classification

The confusion matrices for distinguishing HC from BP, SZ, and DP using the Objective 3-Scale Score feature vectors are presented in Table 3. These matrices visually support the performance metrics summarized in Table 2, showing the distribution of predictions for this quantification approach.

**Table 3:** Confusion matrices (3-Scale Features) for HC vs. BP, HC vs. SZ, and HC vs. DP

HC vs. BP		
Actual\Predicted	HC	BP
HC	45	15
BP	8	12
**HC vs. SZ**		
Actual\Predicted	HC	SZ
HC	47	13
SZ	7	13
**HC vs. DP**		
Actual\Predicted	HC	DP
HC	53	7
DP	8	12

#### Performance of direct NSR area classification

We also evaluated screening using feature vectors directly from the normalized NSR areas. The quantitative results detailing the SVM-based classification performance for distinguishing HC from specific psychiatric groups are summarized in Table 4.

**Table 4:** SVM classification performance using direct NSR area features for HC vs. Specific psychiatric disorders (5-Fold CV balanced Accuracy)

Comparison	Best Parameters Found (Example)	Balanced Accuracy	Sensitivity (Recall for Patient Group)	Specificity (Recall for HC)	Overall Accuracy
HC vs. BP	smote_k_neighbors = 3, svc_C = 1, svc_gamma = 1, svc_kernel: rbf	0.67	0.6500 (BP)	0.6833 (HC)	0.68
HC vs. SZ	smote_k_neighbors = 3, svc_C = 1, svc_degree = 3, svc_kernel: poly	0.65	0.7000 (SZ)	0.6000 (HC)	0.62
HC vs. DP	smote_k_neighbors = 3, svc_C = 1, svc_gamma = 1, svc_kernel: rbf	0.65	0.7500 (DP)	0.5500 (HC)	0.60

#### Detailed precision and recall for direct NSR area classification

The classification metrics for direct NSR area analysis revealed distinct performance across diagnostic groups: For HC vs. BP, precision/recall were 0.85/0.68 (HC) and 0.41/0.65 (BP); for HC vs. SZ, 0.86/0.60 (HC) and 0.37/0.70 (SZ); and for HC vs. DP, 0.87/0.55 (HC) and 0.36/0.75 (DP). These results demonstrate higher precision for HC classifications but variable recall patterns among psychiatric conditions.

#### Detailed confusion matrices for direct NSR area classification

The confusion matrices for distinguishing HC from BP, SZ, and DP using the Direct NSR Area feature vectors are presented in Table 5. These matrices visually support the performance metrics summarized in Table 4, showing the distribution of predictions for this quantification approach.

**Table 5:** Confusion matrices (Direct NSR area Features) for HC vs. BP, HC vs. SZ, and HC vs. DP

HC vs. BP		
Actual\Predicted	HC	BP
HC	41	19
BP	7	13
**HC vs. SZ**		
Actual\Predicted	HC	SZ
HC	36	24
SZ	6	14
**HC vs. DP**		
Actual\Predicted	HC	DP
HC	33	27
DP	5	15

### Comparative analysis of ML screening methods

The analysis of the individual classification reports and confusion matrices reveals a clear distinction in performance between the two proposed quantification approaches. The Objective 3-Scale Score method consistently yielded higher balanced accuracy across all psychiatric disorder classifications (HC vs. BP, HC vs. SZ, HC vs. DP), as shown in Table 6. This method also demonstrated higher overall accuracy and generally higher specificity for Healthy Controls. This indicates that using the empirically derived 3-scale for feature representation results in a more robust and discriminative classification. Conversely, while the Direct NSR Area method provided a continuous measure, its performance in balanced accuracy was generally lower than the 3-Scale Score method, although it showed competitive sensitivity in some cases. The Objective 3-Scale Score method, therefore, proves to be the most effective strategy for psychiatric disorder screening among the evaluated Machine Learning approaches, highlighting that a calibrated quantization of the NSR area can enhance diagnostic precision.

**Table 6:** Classification results of related works (statistics methods)

Methods	Studies	Subject (number and type)	Reported sensitivity	Reported specificity	Sensitivity on our dataset	Specificity on our dataset
M1: NSR score at 0.1 M	Ward et al. [5]	22 HC, 35 SZ	83.0%	77.0%	71.6%	74.0%
M2: NSR score at 0.01 M	Tavares et al. [15]; Lin et al. [16]; Liu et al. [17]	28 HC, 38 SZ, 94 HC, 153 SZ, 40 HC, 61 SZ, 18 BP	13.7–49.2%	85.8–96.8%	34.5%	94.3%
M3: NSR score at 0.001 M	Puri et al. [18]; Smesny et al. [19]	20 HC, 21 SZ, 25 HC, 25 SZ	84.0–90.0%	75.0–76.0%	40.6%	89.9%
M4: Total Score	Puri et al. [20]	26 HC, 27 SZ	77.8%	65.4%	45.2%	89.2%
M5: EC_50_ at 0.1 M and total score	Wang et al. [11]	148 HC, 307 SZ, 179 BP, 127 DP	52.98% in SZ, 60.79% in BP, 53.12% in DP	83.56%	same	same

The overall results from the Objective 3-Scale Score method demonstrate superior performance compared to the direct NSR area method across various psychiatric disorder classifications. This highlights the robust and enhanced potential of these advanced Machine Learning methods for psychiatric disorder diagnostics. Therefore, the comprehensive methodologies introduced in this study prove effective and can be considered as valuable supplementary diagnostic tools for various psychiatric disorders.

## Discussion

This groundbreaking research has unveiled the considerable potential of harnessing the attenuated niacin response, commonly observed in psychiatric patients, for the objective diagnosis of psychiatric disorders. Our initial hypothesis centered on the integration of advanced ML methodologies, specifically the U-net Deep Learning architecture and SVM, to significantly enhance the accuracy and objectivity of NSR detection. This pioneering approach, combining image processing and Machine Learning, holds the promise to revolutionize psychiatric disorder screening.

Previous studies have established NSR as a reliable and objective biomarker for psychiatric disorders, offering a non-invasive and cost-effective diagnostic approach [5, 6, 15–18]. Additionally, research has demonstrated the potential of AI and ML methods in enhancing diagnostic accuracy and efficiency in psychiatry [19], particularly in analyzing complex datasets such as imaging or physiological responses [12, 13, 15, 20]. These strengths provide a solid foundation for further exploration of NSR as a diagnostic tool. However, it is important to highlight the advantages of NSR compared to existing methods, such as clinical assessments and electroencephalography (EEG). NSR is non-invasive, cost-effective, and provides an objective and quantifiable biomarker, making it accessible for use in diverse clinical settings. Moreover, NSR can complement traditional diagnostic tools by offering additional physiological insights, particularly in cases where clinical symptoms are ambiguous or overlap across disorders.

Despite these advancements, several weaknesses and gaps remain. First, existing NSR studies often suffer from variability in testing methods, image acquisition protocols, and analysis techniques, limiting the comparability and generalizability of results [6, 21]. Second, many studies rely on small sample sizes and are confined to specific populations, which restricts their external validity [12, 13, 15, 16]. Third, while traditional statistical approaches have been widely used, the integration of advanced AI and ML techniques in NSR analysis remains underexplored. Finally, previous NSR-based diagnostic tools often require specific devices or settings, which can hinder widespread adoption in clinical practice [21].

A significant achievement of our study is the establishment of the world’s first publicly available NSR dataset. This resource comprises 600 images, each complete with ground truth data, meticulously compiled through rigorous data collection, annotation, and validation processes. This unique dataset paves the way for other researchers to build upon, fostering collaborative advancements in the realm of image-based medical diagnostics. By providing a standardized and open-access resource, we address the lack of consistency in NSR research and enable future studies to replicate and extend our findings. To accelerate collaborative progress, we have designed this open-access dataset as a living resource, a springboard for community-driven expansion. A large-scale multi-center validation study is currently underway, with preliminary recruitment data demonstrating consistent NSR patterns across initial sites. This phased approach allows immediate access to foundational data while addressing scalability through ongoing research.

In addition, we developed an Efficient-Unet (Effb5-Unet, a variant of Unet) model-based system for NSR area detection, achieving an mIOU of 84% and a Dice score to 91%. This system demonstrates exceptional accuracy in identifying skin flushing areas, making a substantial improvement over traditional manual methods and underscores the value of Machine Learning models in enhancing precision within image-based medical diagnostics. Compared to other Deep Learning methods, U-net’s encoder-decoder structure and skip connections proved particularly effective in handling ambiguous boundaries and complex color gradients, ensuring robust and precise segmentation.

Our study also demonstrated the robustness and accuracy of SVM-based classification algorithms, showcasing a progressive improvement across different quantification strategies. By systematically comparing our newly established objective 3-scale derived from segmentation results and the direct use of quantified NSR areas as feature vectors, we revealed a clear hierarchy of performance. Specifically, the Objective 3-Scale Score method consistently yielded the highest diagnostic performance across all psychiatric disorder classifications, outperforming the approach based on direct NSR area values. This highlights the superior discriminatory power of precise, quantized interpretation of the NSR over continuous raw measurements. The SVM, trained with 5-fold cross-validation and enhanced by SMOTE for class imbalance and extensive hyperparameter tuning, pushed its performance to its limits. Compared to existing statistics-based methods (see Table 6), this study clearly illustrates the profound potential of Machine Learning algorithms in the field of psychiatric diagnostics.

By combining the strengths of Efficient-Unet (Effb5-Unet) and SVM, our approach addresses the limitations of traditional methods and provides a scalable, device-independent solution for NSR-based diagnostics. Furthermore, our device-independent framework eliminates the need for specific devices, making it more accessible and adaptable to diverse clinical environments. However, it is essential to acknowledge that NSR is not intended to replace existing diagnostic methods but rather to augment them. Mental health diagnosis remains a complex process that requires the integration of clinical assessments, patient histories, and multiple biomarkers. These findings underscore the transformative potential of AI and ML methodologies in advancing psychiatric diagnostics and pave the way for future innovations in mental health assessment and treatment. Notably, our device-independent framework’s performance suggests ML models may inherently mitigate device-specific biases that challenge manual NSR interpretation, a hypothesis we will rigorously test in the expanded cohort.

## Limitations

While our study established the first standardized NSR dataset with 600 images (*n* = 120), which was a meaningful advancement for this emerging field, we acknowledge two key constraints: (1) the current sample size remains insufficient for deriving definitive clinical thresholds, and (2) external validation across diverse populations is pending. Further clinical validation in diverse populations and settings is also needed to confirm the robustness of our findings. And while our preprocessing pipeline incorporates device-agnostic normalization techniques, the current validation is necessarily limited to single-device data. Future multi-center studies with standardized imaging protocols will be required to fully establish device-independent generalizability. Additionally, the accuracy of our method varied when categorizing patients into different types of psychiatric disorders, indicating an area that requires further research and refinement. Finally, although our AI-based methods show promise, they are not without challenges. The standardization of NSR testing methods and the quality of raw images (e.g., clarity, resolution) remain critical factors that could impact the performance and generalizability of our ML models. And the “black-box” nature of the Deep Learning architecture, despite its high accuracy, may limit clinicians’ trust and willingness to adopt the tool. Moreover, while our device-independent framework is designed to facilitate updates, the ongoing maintenance and retraining of ML models to adapt to new data and clinical practices could pose operational challenges in real-world implementation.

## Implications

The implications of our study are profound, offering significant improvements in the efficiency and accuracy of psychiatric disorder diagnosis. The NSR dataset, combined with the Efficient-Unet (Effb5-Unet) model-based NSR area detection system, contributes to a more automated, objective, and precise diagnostics in psychiatry. Additionally, our robust SVM-based classification algorithm provides a strong foundation for future developments in the field.

Considering our findings and identified limitations, we encourage future studies to expand data collection across diverse demographic groups and strive for enhanced classification accuracy for different types of psychiatric disorders. Continued refinement of the NSR dataset and the development of advanced Machine Learning algorithms will further drive improvements in the diagnostic process within psychiatry.

The discussion section now provides a comprehensive yet easy-to-read summary of the study’s key findings, limitations, and implications. It emphasizes the significant contributions of the research while acknowledging areas for improvement and potential future directions, maintaining a balance between detail and accessibility.

## Future work

While this study demonstrates the potential of integrating AI and ML methodologies for NSR-based psychiatric diagnostics, several avenues for future research can further enhance the field. First, the expansion of the open dataset: increasing the size and diversity of the NSR dataset, including participants from different demographic groups and geographic regions, would improve the generalizability of the findings and ensure broader applicability in clinical settings. Our ongoing multi-center collaboration is currently collecting additional NSR samples across six geographic regions to address this need. Second, development of advanced algorithms: exploring state-of-the-art Deep Learning architectures could further enhance the accuracy and efficiency of NSR area detection and psychiatric disorder classification. Finally, combining NSR data with other biomarkers, such as genetic, neuroimaging, or behavioral data, could provide a more comprehensive diagnostic framework and improve predictive accuracy. Among these, the expansion of the dataset and the integration with multimodal data are particularly promising for future work. A larger and more diverse dataset would enhance the robustness and generalizability of AI models, while multimodal data integration could unlock new insights into the complex interplay of biological and environmental factors in psychiatric disorders. These efforts will establish NSR as part of a next-generation, biologically grounded diagnostic framework for psychiatry.

## Electronic supplementary material

Below is the link to the electronic supplementary material.

Supplementary Material 1

## Data Availability

Data Availability: The dataset can be accessed from: https://doi.org/10.6084/m9.figshare.23614809.v1

## Code availability

https://github.com/Rimsa78/Skin-Flushing-Project.
