# Supplementary material for: An open dataset and machine learning algorithms for Niacin Skin-Flushing Response based screening of psychiatric disorders
Source: BMC Psychiatry. 2025 Aug 4;25:757. doi: 10.1186/s12888-025-07196-2 (PMC12323195; doi:10.1186/s12888-025-07196-2)
Supplement: Supplementary file 1 — Supplementary Material 1 [file 12888_2025_7196_MOESM1_ESM.docx]

**An Open Dataset and Machine Learning Algorithms for Niacin Skin-flushing Response Based Screening of Psychiatric Disorders**

**Supplementary Files**

**Table of Content**

| **Page** | **Content** |
| --- | --- |
| **3-5** | **Supplementary Table 1. A tabular summary of the keywords related to this research area.** |
| **6** | **Supplementary Figure 1. Flowchart of the niacin test.** |
| **7** | **Supplementary Figure 2. Distributions of the normalized areas in the open dataset corresponding to the human’s score.** |
| **7** | **Supplementary Table 2. Mean value, variance, and standard deviation of the distributions of normalized area corresponding to human score.** |
| **7** | **Supplementary Table 3. The thresholds of the normalized area in the proposed 3-points scoring system.** |
| **8** | **Supplementary Figure 3. Mean of the NSR scores at 20-time points for 0.1 M, 0.01 M, and 0.001 M concentrations of AMN.** |

**Supplementary Table 1. A tabular summary of the keywords related to this research area.**

| **Keywords** | **Definition** |
| --- | --- |
| Niacin Skin-Flushing Response (NSR) | When an aqueous solution of methyl nicotinate comes into contact with the skin, it binds to niacin receptors, activating phospholipase A2, which cleaves arachidonic acid (AA) from the phospholipid bilayer of the cell membrane. The free AA is then converted into prostaglandins by cyclooxygenase, leading to vasodilation and the appearance of erythema (redness) on the skin, called niacin skin-flushing response (NSR). In individuals with psychiatric disorders, the NSR pathway is impaired, resulting in an attenuated NSR, which has been reported as a potential biomarker to assist in the diagnosis of psychiatric disorders. |
| Mental Disorders / Psychiatric Disorders (PD) | Psychiatric disorders (PD), also referred to as mental disorders or mental illnesses, encompass a wide range of conditions characterized by significant disturbances in an individual’s cognition, emotional regulation, behavior, or overall psychological functioning. The diagnosis of PD is based on standardized criteria outlined in diagnostic manuals such as the DSM-5 (Diagnostic and Statistical Manual of Mental Disorders) or ICD-11 (International Classification of Diseases), and treatment often involves a combination of pharmacological, psychological, and psychosocial interventions. The PD examined in this paper mainly consist of depression, bipolar disorder, and schizophrenia. |
| Artificial Intelligence (AI), Machine Learning (ML) and deep learning (DL) | Artificial Intelligence (AI) refers to the development of computer systems capable of performing tasks that typically require human intelligence, such as learning, reasoning, problem-solving, perception, and decision-making. AI leverages algorithms, machine learning (ML), and deep learning (DL) to analyze vast datasets, identify patterns, and make predictions or classifications. ML, a subset of AI, focuses on training models to improve performance through experience, while DL, a specialized form of ML, uses neural networks to process complex data structures. |
| U-net | U-Net is a specialized deep learning architecture designed for image segmentation tasks, particularly in biomedical imaging. It features a symmetric encoder-decoder structure, where the encoder captures contextual information through convolutional and pooling layers, while the decoder reconstructs precise spatial details using up-convolutional layers. A key innovation of U-Net is its skip connections, which bridge the encoder and decoder to preserve fine-grained details and improve segmentation accuracy. Originally developed for medical image analysis, U-Net has demonstrated exceptional performance in tasks requiring pixel-level classification, such as identifying regions of interest in medical scans. Its efficiency and robustness make it a widely adopted model in image-based diagnostics and beyond. |
| Manual Score | The manual scoring of NSR-induced flushing is conducted based on a 4-point scale, with the criteria defined as follows: 0 = No erythema; 1 = Incomplete erythema; 2 = Complete erythema within the niacin stimulation area; 3 = Erythema extending beyond the niacin stimulation area. |
| Open Dataset | A publicly accessible database containing NSR test photographs, manual scoring results, and AI learning outcomes for both psychiatric disorder patients and healthy controls. |
| Detection of the NSR Area | Detection of the NSR Area refers to the precise identification of niacin-induced skin-flushing regions using advanced image processing techniques. Leveraging a deep learning architecture, the methodology enables objective and accurate segmentation of erythema (flushing) areas in high-resolution images. This process involves analyzing color differences between the NSR area and normal skin to generate binary segmentation masks, assigning pixel-level labels to distinguish the target regions. The approach addresses challenges such as ambiguous boundaries and complex color gradients, achieving high precision with metrics like mIoU and Dice score. This automated detection system eliminates reliance on manual methods, providing a scalable and device-independent solution for NSR-based diagnostics in psychiatric disorders. |
| Quantification of NSR (*A_norm_*) | The subject's arm is marked with a uniformly sized label containing an identification number, which serves as a normalization standard in machine learning. The normalized area value is obtained by calculating the ratio of the Detected NSR Area to the area of the label. |
| Support Vector Machine (SVM) | Support Vector Machine (SVM) is a supervised machine learning algorithm used for classification and regression tasks. It works by identifying an optimal hyperplane that maximizes the margin between data points of different classes in a high-dimensional feature space. SVM employs kernel functions (e.g., linear, radial basis function) to transform non-linearly separable data into a separable form. Its robustness in handling high-dimensional data and ability to generalize well with limited samples make it particularly effective for complex classification problems. SVM is widely applied in fields such as bioinformatics, image recognition, and text classification, offering high accuracy and stability in predictive modeling and pattern recognition tasks. |
| Mean Intersection Over Union (mIOU) | Mean Intersection over Union (mIoU) is a performance metric widely used in image segmentation tasks to evaluate the accuracy of predicted regions compared to ground truth. It calculates the ratio of the overlapping area (intersection) to the total combined area (union) between predicted and actual segments, averaging this value across all classes or regions. mIoU ranges from 0 to 1, with higher values indicating better segmentation accuracy. It is particularly effective for assessing models in tasks with imbalanced class distributions or complex object boundaries, making it a standard metric for evaluating segmentation performance in computer vision and medical imaging applications. |
| Dice Score | Dice Score, also known as the Dice Similarity Coefficient (DSC), is a metric used to evaluate the similarity or overlap between two sets of data, commonly employed in image segmentation tasks. It ranges from 0 to 1, where 1 indicates perfect overlap and 0 indicates no overlap. The Dice Score is calculated as twice the intersection of the predicted and ground truth regions divided by the sum of their areas. It is particularly useful for assessing the accuracy of segmentation algorithms in medical imaging, providing a robust measure of spatial agreement between predicted and actual regions of interest. |
| Sensitivity | Sensitivity is a statistical measure that evaluates the ability of a test or model to correctly identify true positive cases, particularly in binary classification tasks. It is calculated as the ratio of true positives to the sum of true positives and false negatives (Sensitivity = TP / (TP + FN)). High sensitivity indicates that the test effectively detects the condition of interest, minimizing false negatives. |
| Specificity | Specificity in medical contexts refers to the ability of a diagnostic test to correctly identify individuals without the disease, or true negative rate. It is calculated as the proportion of true negatives (correctly identified healthy individuals) among all individuals who do not have the condition. |
| Accuracy | In medical research, accuracy refers to the degree to which a diagnostic test or measurement correctly identifies or classifies true positive and true negative cases within a given population. It is calculated as the ratio of the sum of true positives and true negatives to the total number of cases tested, providing an overall measure of the test's correctness. High accuracy indicates that the test reliably distinguishes between affected and unaffected individuals, minimizing both false positives and false negatives. This metric is critical for evaluating the effectiveness of diagnostic tools, enabling clinicians and researchers to assess the reliability and validity of medical tests in clinical practice and research settings. |

Note: The keywords are ordered according to their sequence of appearance in the manuscript.


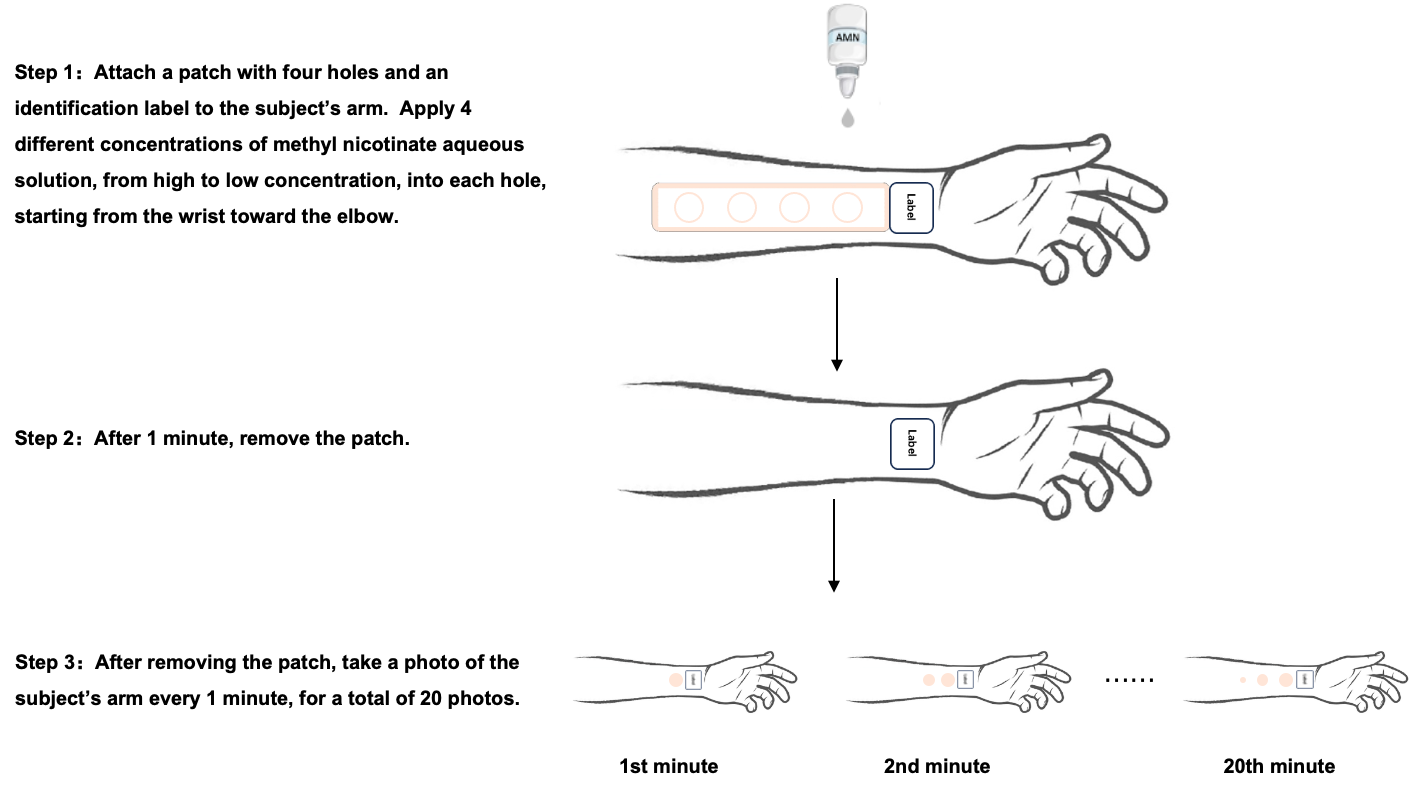


**Supplementary Figure 1. Flowchart of the niacin test.** A brief introduction to the population undergoing the niacin test is as follows:

**Healthy Controls:** The healthy control group (HC) comprised 154 individuals with no prior history of psychiatric illness. This group included 41 males and 113 females with an average age of 34.67 (8.8) years, average weight of 60.98 (11.2) kilograms (kg), and average height of 164.47 (7.3) centimeters (cm). Their average Body Mass Index (BMI) was 22.46 (3.3) kg/m². Approximately 36.37% (56 individuals) reported alcohol use, while approximately 7.14% (11 individuals) reported nicotine use.

**Schizophrenia:** This group consisted of 311 patients diagnosed with SZ, including 172 males and 139 females. The average age was 40.50 (13.0) years, average weight 71.04 (13.6) kg, and average height 167.43 (8.5) cm. Their average BMI was 25.36 (4.4) kg/m². Approximately 16.61% (52 individuals) reported alcohol use, and approximately 3.19% (10 individuals) reported nicotine use.

**Bipolar Disorder:** This group encompassed 184 BP patients, with 117 males and 67 females. They had an average age of 37.75 (14.1) years, average weight of 74.71 (14.2) kg, and average height of 169.11 (8.0) cm. The average BMI was 26.08 (3.8) kg/m². Approximately 19.02% (35 individuals) reported alcohol use, and approximately 8.15% (15 individuals) reported nicotine use.

**Depression:** This group involved 137 patients diagnosed with DP, including 45 males and 92 females. Their average age was 42.80 (16.1) years, average weight of 65.88 (12.2) kg, and average height of 164.60 (8.5) cm. The average BMI was 24.30 (3.8) kg/m². Approximately 10.22% (14 individuals) reported alcohol use, and approximately 7.30% (10 individuals) reported nicotine use.


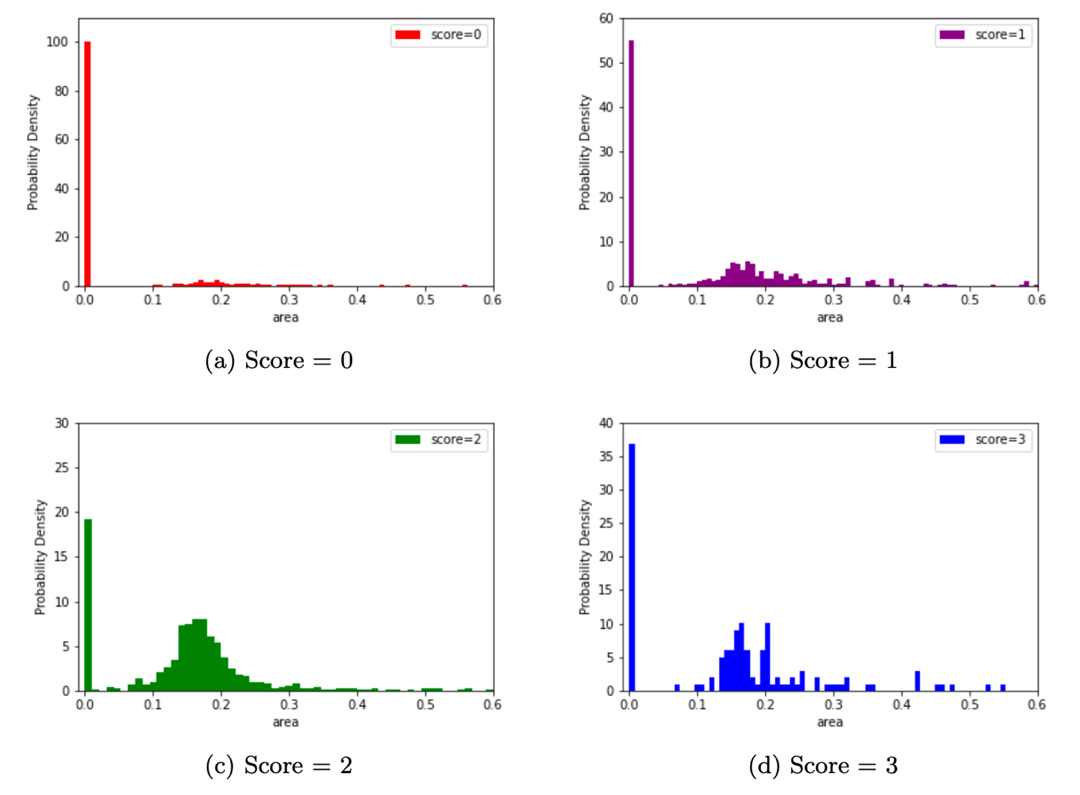


**Supplementary Figure 2. Distributions of the normalized areas in the open dataset corresponding to the human’s score.** The x-axis represents the normalized area size.

**Supplementary Table 2. Mean value, variance, and standard deviation of the distributions of normalized area corresponding to human score.**

| Score | Mean value | Variance | Standard deviation |
| --- | --- | --- | --- |
| 0 | 0.0648 | 0.0229 | 0.1515 |
| 1 | 0.1535 | 0.0363 | 0.1907 |
| 2 | 0.1661 | 0.0217 | 0.1477 |
| 3 | 0.1665 | 0.0291 | 0.1706 |

**Supplementary Table 3. The thresholds of the normalized area in the proposed 3-scale scoring system.**

| Score | Lower bound | Upper bound |
| --- | --- | --- |
| 0 | 0 | 0.1091 |
| 1 | 0.1091 | 0.1598 |
| 2 | 0.1598 | - |

The distributions of the normalized areas corresponding to different scores are distinctly illustrated in Supplementary Figure 2. The mean values, variances, and standard deviations of these distributions are thoroughly tabulated in Supplementary Table 1. Given the considerable variance and overlap among the distributions, a 3-point scoring system was formulated, with its thresholds detailed in Supplementary Table 2.


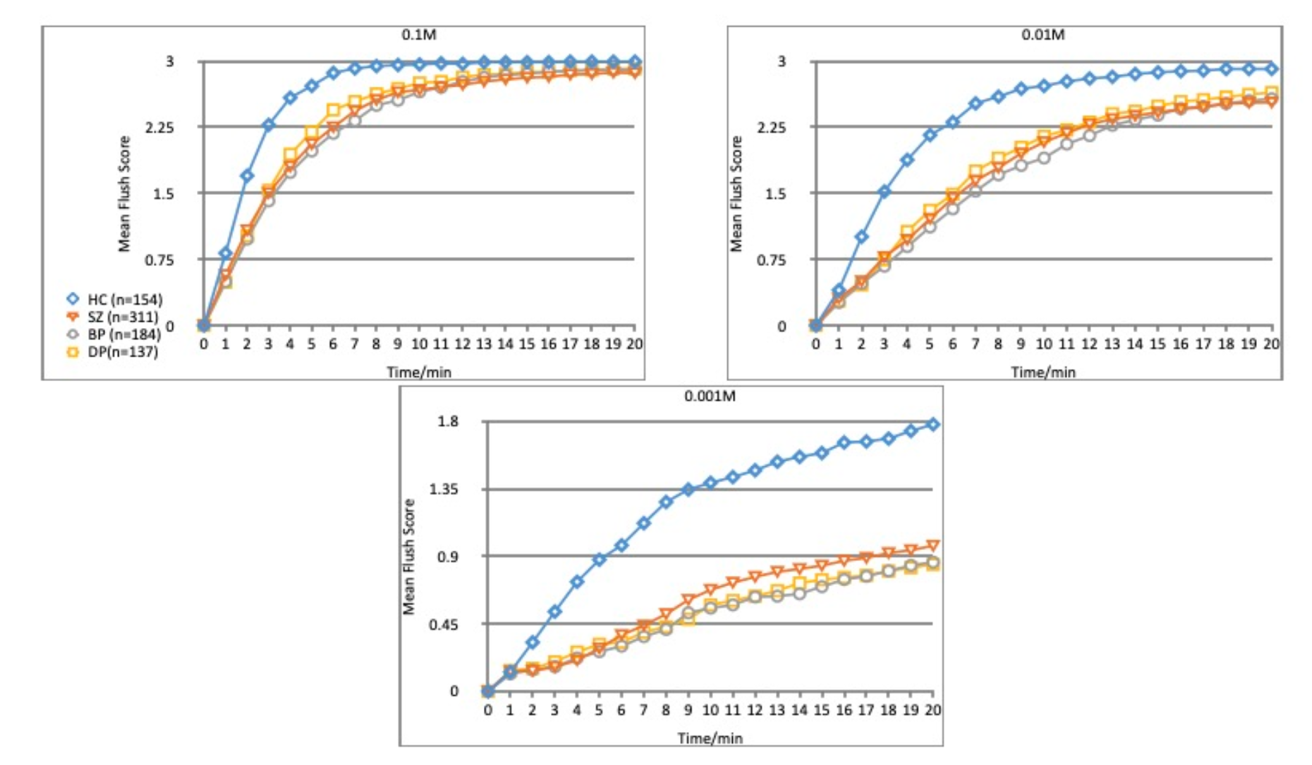


**Supplementary Figure 3. Mean of the NSR scores at 20-time points for 0.1 M, 0.01 M, and 0.001 M concentrations of AMN.**

Supplementary Figure 3 elucidates the NSR as estimated by manually annotated 4-scale scoring, presenting the mean NSR score across different groups. The contrast between the healthy control (HC) group and psychiatric groups is pronounced, although the responses within the psychiatric groups exhibit similarities. Notably, a concentration of 0.0001 M AMN, which is excluded here, was ineffective at inducing NSR in the majority of subjects. Our findings reveal a slower evolution of NSR areas in psychiatric patients compared to healthy individuals, with minimal variance among the disorders being assessed.
